# Life History Consequences of the Prevalence of Aggressive Males Carrying Costly Weapons

**DOI:** 10.1002/ece3.73790

**Published:** 2026-06-08

**Authors:** Neha Pandey, Przemysław Piekarczyk, Radosław Gmyrek, Norbert Czyżewski, Paulina Łukaszyk, Agnieszka Szubert‐Kruszyńska, Sebastian Chmielewski, Tom C. Cameron, Jacek Radwan

**Affiliations:** ^1^ Evolutionary Biology Group, Faculty of Biology Adam Mickiewicz University Poznań Poland; ^2^ Department of Biological and Environmental Science University of Jyväskylä Jyväskylä Finland; ^3^ School of Life Sciences University of Essex Colchester UK

## Abstract

Sexually selected weapons used in intra‐sexual competition for mates are among the most striking animal features, but how their evolution affects life history traits closely correlated with fitness, and hence species' evolutionary trajectories, is not well understood. Here, we selected for or against male morphs carrying a lethal weapon in a male‐dimorphic mite *Sancassania berlesei*, and investigated how life histories evolve in populations with high vs. low proportion of weaponized, aggressive males called fighters and non‐weaponized, non‐aggressive males called scramblers. After 25 generations of experimental evolution, females from fighter‐selected lines showed higher early‐life fecundity compared to females from lines selected for non‐aggressive scrambler males. Furthermore, both sexes matured earlier in fighter‐selected lines compared to scrambler‐selected ones. Larvae‐to‐adult survivorship was not affected by such selection treatment. Finally, we investigated whether adult survivorship under temperature stress was influenced by such selection treatment, and we found no difference between fighter‐selected and scrambler‐selected lines. Our results demonstrate that composite selection pressures resulting from the prevalence of costly intra‐sexual aggression lead to an increase in key components of fitness, with likely consequences for population dynamics. However, we found no evidence that the response to such selection affects how individuals cope with environmental challenge.

## Graphical abstract

Using a soil mite system with dimorphic males, that is, aggressive, weaponized fighters and benign scramblers, we experimentally selected populations for higher proportion of fighter males for 25 generations. Populations with higher proportion of fighter males evolved faster juvenile development and higher early‐life fecundity. However, this evolution did not improve the survival of fighter populations in response to heat stress.

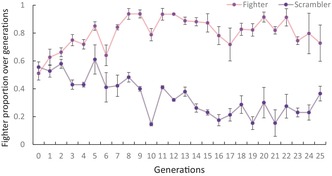

## 1 Introduction

Sexual selection is a powerful evolutionary process favouring traits used in competition for mates and access to their gametes. The most striking examples of such traits are weapons used in direct competition and exaggerated ornaments used to attract mates, but sexual selection may also favour other traits, such as high body mass enabling competition for resources or high mobility required for mate searching (M. B. Andersson 1994). However, sexually selected traits are often costly to produce and/or maintain, and these costs make them non‐independent from other fitness components competing for the same set of resources or sharing molecular pathways (Husak and Lailvaux 2014; Radwan et al. 2015). On the one hand, this non‐independence may result in fitness trade‐offs, with increased investment in sexually selected traits associated with decreased survival (Hunt et al. 2004; Okada et al. 2011; Johnston et al. 2013; Nandy et al. 2013), immune function (Kawecki 2020) or increased development time (Garlovsky et al. 2022). Furthermore, sexual selection acting on males may simultaneously act directly or indirectly (via pleiotropic effects) on traits expressed in females, which may lead to decreased female fitness if the optimal trait values differ between sexes (Rice and Chippindale 2001; Bonduriansky and Chenoweth 2009; Pennell and Morrow 2013). For example, in *Callosobruchus maculatus* beetles, females which evolved under enforced monogamy (and thus reduced sexual selection) had increased mortality compared to females evolved under polygamous system (Maklakov et al. 2007), whereas in broad horned beetles natural selection towards increased female fitness selected against elaboration in male sexual traits (Okada et al. 2021). On the other hand, sexual selection may have positive effects on non‐sexually‐selected fitness components by enhancing selection for adaptive variants, or against deleterious variants. This is because costliness of sexually selected traits makes them condition‐dependent, with condition reflecting organismal efficiency in acquiring and utilising resources (M. Andersson 1986; Rowe and Houle 1996). For example, such relationship was argued to underlie positive association between peacock train elaboration and progeny survival (Petrie 1994) or the positive effect of selection for the size of sexually selected male weapon in a mite *Rhizoglyphus echinopus* on female fecundity (Buzatto and Clark 2020). The balance between life‐history trade‐offs and benefits associated with sexual selection is likely to impact population viability and chances to persist under environmental challenge (Kokko and Brooks 2003; Candolin and Heuschele 2008). For example, high male investment in costly sexually selected traits may increase chances of species extinction (Bro‐Jørgensen 2014), but on the other hand, intense sexual selection may positively affect population resilience to stress (Berger and Liljestrand‐Rönn 2024; Godwin et al. 2020; Iglesias‐Carrasco et al. 2024), by purging inbreeding depression and thus making populations less sensitive to environmental stress (Fox and Reed 2011).

Although a meta‐analysis of experimental evolution studies that manipulated the opportunity for sexual selection, mostly by altering mating systems, concluded that on average sexual selection results in an improvement in fitness‐related traits (Cally et al. 2019), however the effect size was low, echoing a non‐significant relationship between sexually selected traits associated with male attractiveness and fitness traits (Prokop et al. 2012). The degree of alignment between natural and sexual selection may have a key impact on population productivity (Berger et al. 2016) and resilience to environmental change (Candolin and Heuschele 2008; Łukasiewicz et al. 2023). However, the alignment may vary depending on the ecological context of sexual selection or the type and genetic architecture of sexually selected traits (SSTs) (Long et al. 2012; Rowe and Rundle 2021), and data on the relationship between sexually selected traits and naturally selected fitness components from a broader range of species and contexts are needed to understand this variation.

Male weapons serving in intra‐sexual competition are an excellent system to investigate the alignment between sexually selected and fitness‐related traits because they are among the most intra‐specifically variable traits (Emlen 2008). Furthermore, their cost of production and maintenance makes them condition‐dependent (e.g., Emlen 1997; Kotiaho et al. 2001), which, as argued above, could indirectly select for other condition‐dependent fitness traits, either positively via enhancing selection for beneficial variants (Parrett et al. 2019; Buzatto and Clark 2020), or negatively due to trade‐offs (Johnston et al. 2013; Bro‐Jørgensen 2014). Interestingly, the trade‐off may involve sexually antagonistic selection even when the weapons are male‐limited in expression (Harano et al. 2010; Plesnar‐Bielak et al. 2014). For example, in the bulb mite (*Rhizoglyphus robini*), where males are dimorphic for expression of a weapon in the form of thickened and sharply terminated third pair of legs, lines selected for higher expression of weaponized fighter morphs showed reduced female fecundity compared to lines selected for unarmed scrambler morph (Plesnar‐Bielak et al. 2014). However, in a congener *R. echinopus*, the reversed relationship was found: fighter male lines selected for thicker third pair of legs were more fecund than unselected controls (Buzatto and Clark 2020), indicating that selection on this condition‐dependent weapon (Tomkins et al. 2011) might have led to increased condition, and thus higher fitness in both sexes. Thus, even though the selection protocol differed between the two studies (weapon presence/absence in *R. robini* vs. weapon size in *R. echinopus*), these varying outcomes suggest that even for a homologous sexually selected trait, its coevolution with fitness traits can vary.

In addition to traits genetically correlated to the weapon, the prevalence of aggressive males in a population may affect selective environment in which life‐history traits evolve, with consequences on population dynamics (Porwal et al. 2025; Pandey et al. 2026). For example, increased mortality may select for early maturation (Kozłowski et al. 2026). Furthermore, male fight‐related mortality alters sex ratio, and thus the level of male harassment of females, in turn modifying selection on female life‐histories (Tilszer et al. 2006). Experimental evolution is an excellent tool to understand overall consequences of the presence of aggressive, armed males in a population (Parrett et al. 2022; Porwal et al. 2025).

Here, we investigated life‐history correlates of the prevalence of a sexually selected weapon in a population of *Sancassania berlesei*, one of several species of acarid mites in which sexually selected male dimorphism has been described (Radwan 2009). Interestingly, acarid species differ in morph determination. While condition‐dependence seems a shared feature across species (Radwan 1995; Smallegange 2011; Tomkins et al. 2011), in *S. berlesei* and in *R. echinopus* (but not in *R. robini*), the fighter morph is pheromonally suppressed in large populations, such that two morphs only co‐occur at intermediate population sizes (Radwan 1993). Such pheromonal sensitivity is adaptive as female monopolisation by fighters is only possible in small populations, whereas in large populations, fighters pay mortality costs of aggression (Radwan 1993; Radwan et al. 2014; Michalczyk et al. 2018). Conversely, morph determination is characterised by a large additive genetic component in *R. robini* (Parrett et al. 2023), while no such strong additive effect was found in *R. echinopus* or *S. berlesei* (Radwan 1995, 2001; Buzatto et al. 2012b). It is nevertheless possible to select for morph proportion in the latter species by selecting for the threshold of sensitivity to pheromones (Unrug et al. 2004) or for the threshold of sensitivity of the condition at which the fighter morph is expressed (Buzatto et al. 2012a). In *S. berlesei*, it was earlier demonstrated that starting at a population density at which pheromone concentration allows expression of approximately even proportion of both fighter and scrambler morphs, one may readily increase fighter or scrambler proportion by selecting as sires of the next generation males that express the desired morph, without concomitant change in condition itself (Unrug et al. 2004). Here, we used similar selection to increase or decrease the proportion of fighters in replicate experimental evolution populations and maintained the altered proportions for 25 generations. We then investigated the consequences of increased or decreased proportion of sexually selected fighters for three major life‐history traits: larvae‐to‐adult development time and survivorship, female early fecundity, and adult survivorship under environmental challenge. The overall consequences of morph prevalence for fitness‐related life history traits are likely to be shaped by a complex interplay of several mechanisms. For example, if selection for the fighter morph, and the consequent high prevalence of aggressive interactions in the population increased purifying selection against maladaptive variants, we expect all or most of the fitness‐related traits and resilience to stress to improve compared to the alternative treatment. However, if selection for the male weapon and, indirectly, for fighting efficiency caused evolutionary response in sexually antagonistic loci, we expected female fecundity to be lower in fighter‐selected lines. Furthermore, increased male mortality in populations with high fighter prevalence might also directly select for early maturity, in which case we expected males to mature earlier in fighter‐selected lines.

## 2 Methods

### 2.1 Maintenance and Selection

Our experimental design consisted of six replicates divided between two treatments mass‐selected towards fighter morph or scrambler morph. The mites came from stock population collected from a poultry farm in Dluga Goslina, Wielkapolska, Poznań, Poland in the year 2022. Species designation (according to Hughes 1976) was validated by sequencing COII fragment, which proved identical to *S. berlesei* accession NC_024637. The culture was then maintained in the lab in an overlapping generation cycle at large population size (≫500 adults) at 23°C; humidity was maintained at > 90% and powdered yeast was given as a food. We first determined population density at which both morphs are expressed at approximately equal proportion by placing 20, 30, 40, 50, 60, 70 and 80 larvae or protonymphs (in absence of larvae) with three replicates each in small plastic containers (~2.5 cm diameter; ~2 cm height). These containers had plaster‐of‐Paris bottom (~1 cm) and plastic plug with holes secured with cotton ball for gas exchange, and were provided with 2 spoons (spoon dimensions: ~9.6 × ~4.8 mm) of yeast. Pheromone‐sensitive window occurs at early tritonymphal stage, and we determined that 50 larvae result in 46% fighter emerging on average (Figure S1). To start the selection protocol, we placed 50 larvae/protonymphs from the stock population in each of plastic containers, and randomly assigned the containers to three replicate lines of one of the two treatments: scrambler and fighter. To ensure we had enough individuals of the desired morph within each line, we set up six containers per line. Upon emergence of adults, in fighter treatment we selected 40 fighter males and 80 females at random from the six containers containing mites from the same selection line, and in scrambler treatment we accordingly selected scrambler males and females. We then left selected males and females in a new plastic container for ~3 days and after this time moved 40 females to a sterile container to oviposit for 24 h which was used to start next generation (Figure S2). Close to adult emergence time the containers were checked and the undesired morph was removed. We could not exclude the possibility of the undesired males having mated before they were removed; however, there is strong last male precedence in this species, such that most of the sperm deposited by a male is likely to be displaced by that from consecutive male partners before they have a chance to fertilise any eggs (Radwan 1991). Since females would typically remate several times a day, and there is ca. 24 h between fertilisation and oviposition, vast majority of eggs would be fertilised by sperm of a selected male. To start next generation, we again placed 50 larvae/protonymps (in absence of larvae) and followed steps describe above for the first generation. This design, while directly selecting for or against the weapon, also changes the nature of sexual selection. This is because males competed for the access to 80 females for ~3 days, and this competition included lethal fights which were necessarily becoming rare in scrambler‐selected lines, but more common in fighter lines as selection progressed. This design mimicked what happens in natural population expressing different levels of sexually selected aggression and allowed us to assess the total effect of the presence of weapons in a population. We carried out this selection for 25 generations, recording proportion of morphs in each replicate, after which we carried out fitness assays.

### 2.2 Fitness Assays

To estimate larvae‐to‐adult development time, we collected 130 mated females from each selected line (after population expansion from generation 25) and allowed them to lay eggs in a narrow time window of 7 h. During this period, females were provided with ample food and moisture and housed in large plastic dishes (length ~9.5 cm; width ~7 cm; height ~4.5 cm). After 7 h all the females were removed from the containers. The dishes were left with food, and a small amount of water was sprayed to allow the eggs to hatch over the next 4–5 days. Once a sufficient number of larvae had hatched, we established three replicates for each selection line, placing 50 larvae randomly in each replicate. We started development time checks from 76 h after larvae collection, and checks were performed every 12 h until all adults had emerged, with the last two checks at 23‐ and 24‐h interval. We also collected data on larvae‐to‐adult survivorship from this assay.

Early female fecundity was measured at the fourth day after emergence, that is, the time window at which fecundity was relevant to female fitness during experimental evolution. To estimate female fecundity, we mated selected females with males from stock culture rather than from their own lines to separate it from the possible effect of male morph/treatment on female fecundity. We collected 50 freshly emerged virgin females from each selected line (after 20th generation) and paired them with 50 males from stocks that were ~2.5–3 days old. Males and females were housed together in plastic containers with ample food and enough moisture for ~3 days. Subsequently, 30 females were randomly selected from each container and housed individually in vials containing sufficient food. Females were allowed to oviposit for 24 h in vials after which eggs were counted.

To estimate adult survivorship under heat treatment, we collected 30 females and 30 males from each selection line (after 20th generation), and housed these ~2 days old adults in vials individually. The adults were then exposed to 40°C for 90 h, and their mortality after the exposure was recorded. These stress parameters followed Pandey et al. (2026), who selected them based on pilot experiments and showed that it can cause significant mortality and perturbations to population dynamics.

### 2.3 Statistics

Statistical analyses were done with the glmmTMB package (Brooks et al. 2017) implemented in R studio (Posit) using R version 4.5.0 (R Core Development Team 2025). To analyse morph proportions across generations we linearized them using logit transformation. The model contained line as a random factor. Fitness components were analysed with general (fecundity, development time) or generalised (proportion survived) mixed models, the latter using binomial error distribution and logit link function. In these models, morph treatment was entered as a fixed factor, and a line and a replicate nested within the line as random factors (except for survival data where a replicate represented a single datapoint). Non‐significant interactions (*p* > 0.05) were removed from models. Model assumptions were checked using diagnostic plots, which for generalised models were produced by *DHARMa* (Hartig 2016).

## 3 Results

**FIGURE 1:**
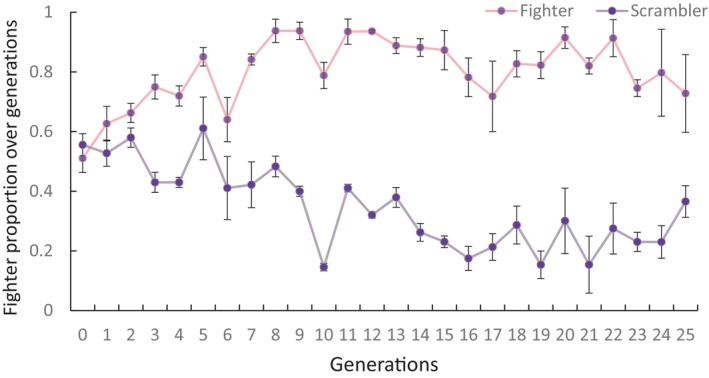
Proportion of fighter morphs in the fighter and scrambler selection lines over 25 generations. Error bars around the mean are standard error.

Our selection experiment was successful in changing proportion of males (Figure 1; selection × generation interaction *Z* = −5.585; *p* < 0.001, Table S1). Around generation 10, fighter‐selected lines reached a proportion of ca. 0.8 males being fighter morphs, whereas scrambler selected lines reached similar proportion of scrambler morphs. After generation 10, the proportion of selected morphs ceased to further increase (Figure 1).

**FIGURE 2:**
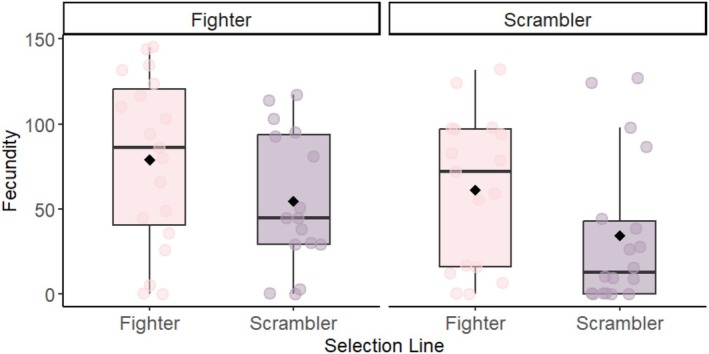
Early life fecundity in the fighter and scrambler selection lines, when females were paired with males from the stock population. The upper header represents the male type from the stock population.

Our manipulation of morph proportions in experimental evolution lines resulted in divergence between the treatments in key life‐history traits. Early life fecundity of females mated with males from the unselected stock population after generation 20 was significantly higher in fighter lines compared to scrambler lines (Figure 2; *Z* = −2.506, *p* = 0.012, Table S2), whereas the morph of the male partner did not have a significant effect (*Z* = −1.848, *p* = 0.065).

**FIGURE 3:**
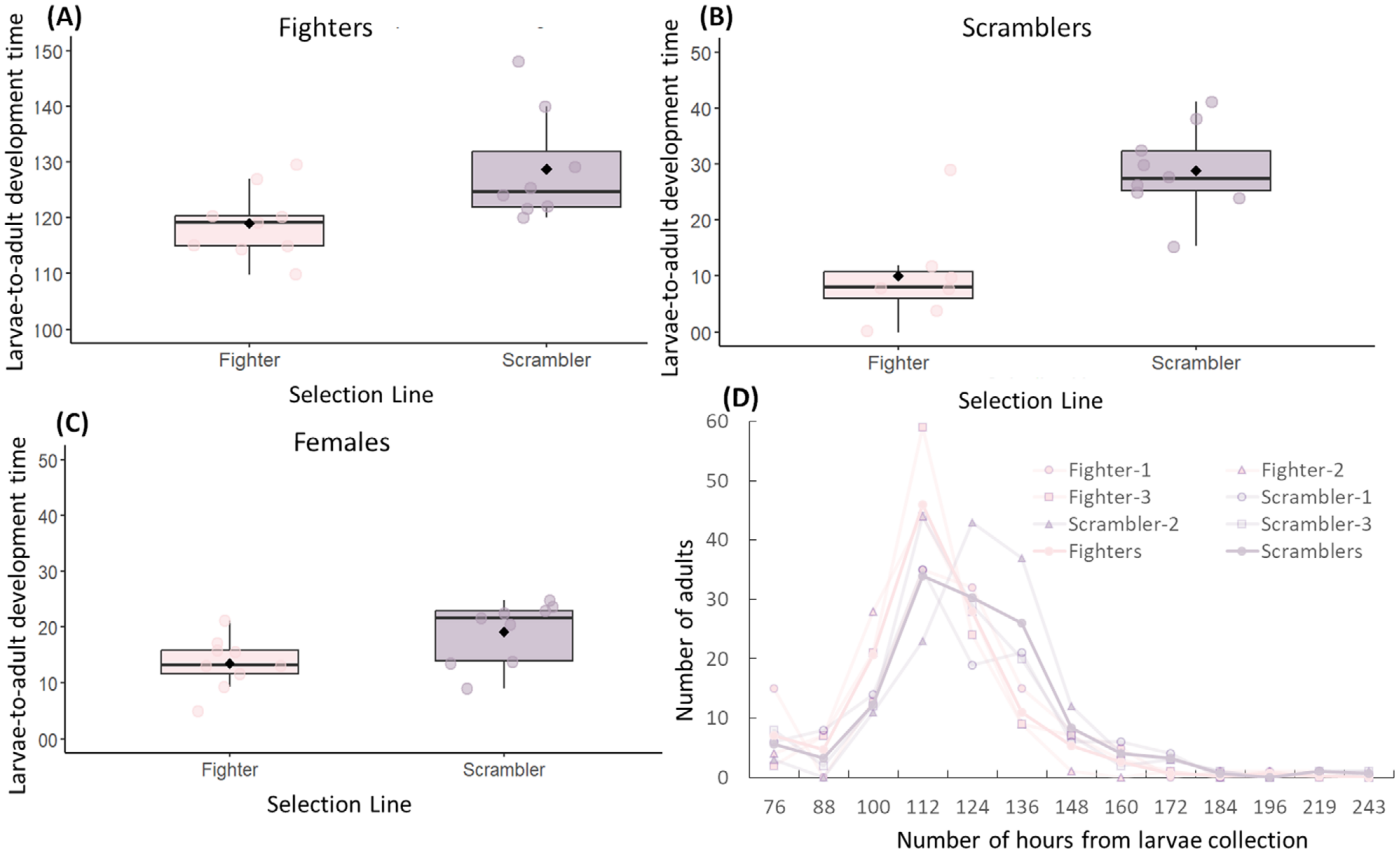
Larvae‐to‐adult development time in the fighter and scrambler selection lines at generation 25. (A) Development time for fighter male morphs, (B) development time for scrambler male morphs, (C) development time for females. (A–C) represent average development time across three replicates (D) development time in all replicate populations (treatment assignment explained in legends).

**FIGURE 4:**
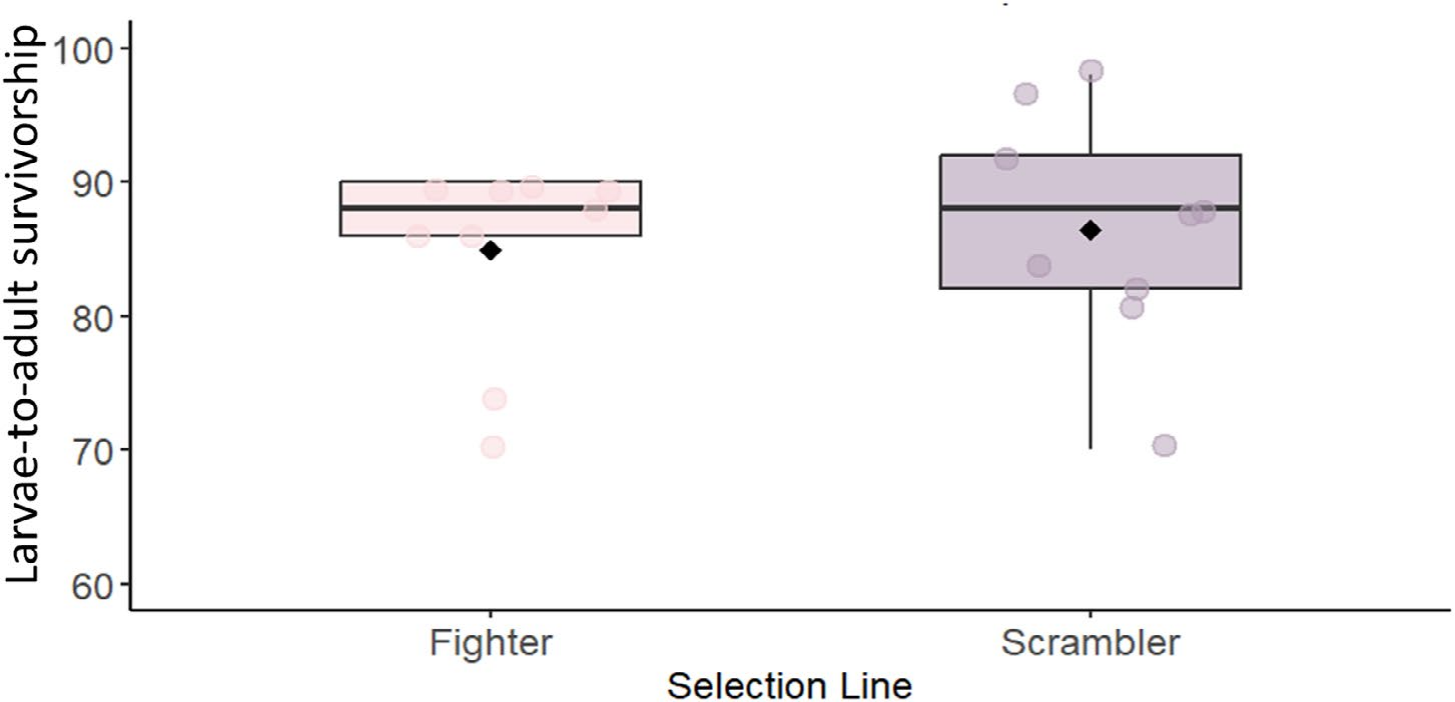
Larvae‐to‐adult survivorship in the fighter and scrambler selection lines after 25 generations of selection.

**FIGURE 5:**
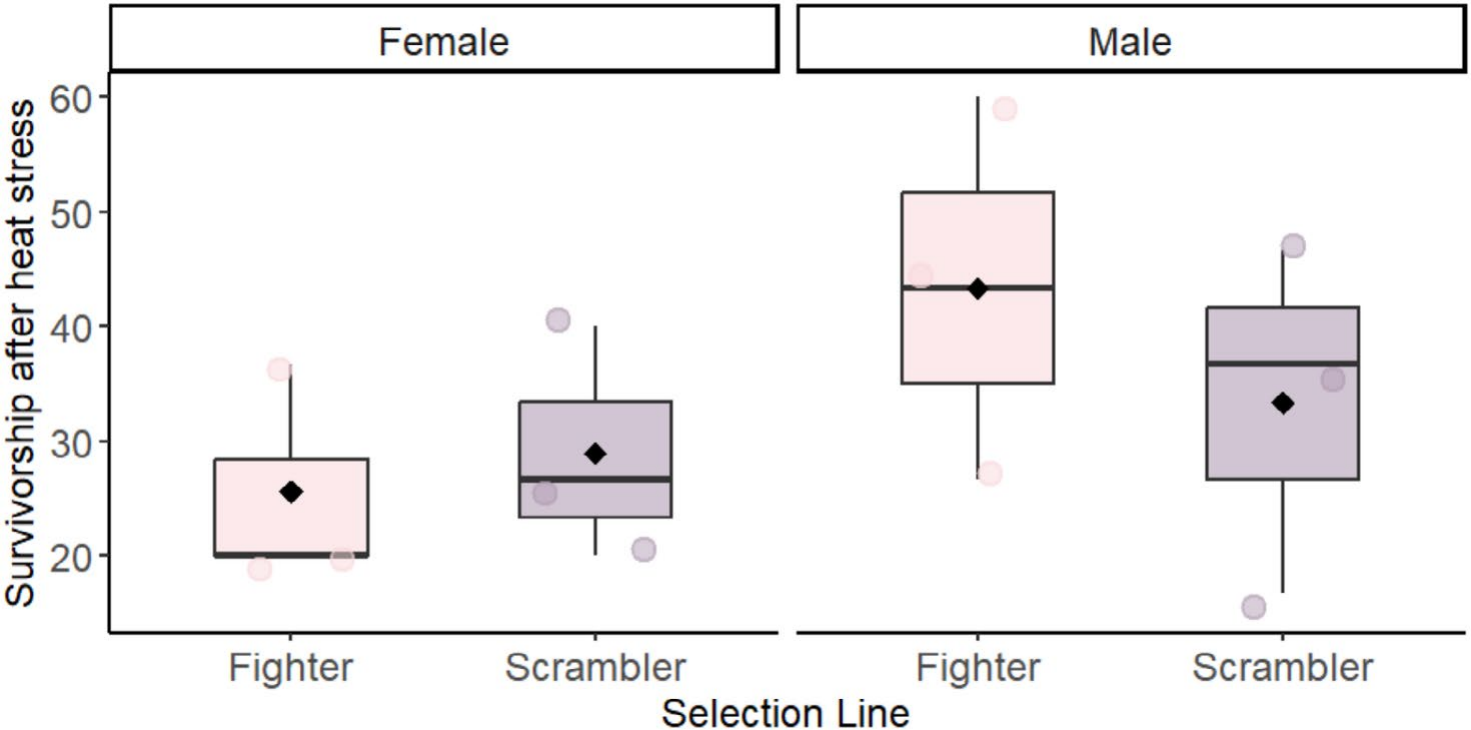
Average adult survivorship in fighter and scrambler selection lines after heat exposure for males and females (specified in headers).

Larvae‐to‐adult development time was the shortest for females, followed by fighters and scramblers (Figure 3). The development time was longer in mites coming from scrambler selected lines compared to fighter selected ones (*Z* = 2.04, *p* = 0.041, Table S3) after accounting for morph and sex differences. Survival from larvae‐to‐adult did not differ between our treatments (Figure 4, Table S4). Finally, there was no significant difference in adult survival between treatments after exposure to heat stress, but males survived for longer than females (Figure 5, Table S5).

## 4 Discussion

Evolution of costly traits mediating male success in reproductive competition is likely to have consequences for evolution of male and female life histories, with consequences for population viability (Candolin and Heuschele 2008; Berger et al. 2016; Łukasiewicz et al. 2023). Here, we investigated the effect of the prevalence of sexually selected male weapons in a population on early life female fecundity, development time, juvenile survival and adult survivorship under environmental stress. We found that in populations selected for higher prevalence of fighter morphs, development time shortened for both sexes, while female fecundity increased. There was no significant effect on juvenile survival. We note that we have not estimated lifetime fecundity or survival, but we chose to measure fecundity and survival relevant to fitness during 25 generations of evolution, where fecundity at Day 4 (and survival till that time) determined fitness. Furthermore, in species with overlapping generations early fecundity contributes most to lifetime fitness in competitive population dynamics (Fisher 1930; Caswell 2001; Plaistow et al. 2007). Thus overall, the effect of increased prevalence of a costly weapon in the population on fitness‐associated life history traits was generally positive, suggesting that enhanced selection favouring beneficial variants (or disfavouring deleterious ones) prevailed over any negative effects that might have arisen due to fitness trade‐offs resulting from male investment in weapons and costly aggressive behaviours.

One important trade‐off involves sexually antagonistic pleiotropy reported in other species with weaponized males, including other acarids (Harano et al. 2010; Plesnar‐Bielak et al. 2014). In *R. robini*, selection protocol similar to ours led to decreased female fecundity (Plesnar‐Bielak et al. 2014), the opposite of our results. The two species differ in the mode of male morph determination, being highly heritable in *R. robini*, but predominantly environment‐cued in *S. berlesei* (Radwan 1995; Tomkins et al. 2004; Parrett et al. 2023). In *R. robini* morph determination maps to a large region of reduced recombination (Chmielewski et al. 2025). Such a region can host sexually antagonistic loci (Jordan and Charlesworth 2012) or accumulate deleterious variants (Carpentier et al. 2022; B. Charlesworth and Charlesworth 2000). It seems therefore plausible that sexually antagonistic and/or deleterious variants residing in this morph‐determining supergene contributed to fitness costs observed in *R. robini* females. Interestingly, similarly to *S. berlesei*, male morph is mostly environment‐cued and condition‐dependent in *R. echinopus*. Also similarly to our results, selection for male weapon size led to a correlated increase in female fitness (Buzatto and Clark 2020). The contrasting results for *R. robini* on one hand, and the other two acarids on the other, highlight the possibility that the direction of the relationship between sexual selection and life‐history traits may depend on the genetic architecture of SSTs.

While our results indicate that in *S. berlesei* sexual antagonism (or other trade‐offs) does not prevail over positive effects of selection on male weapons and associated aggressive interactions on fitness‐related traits, these positive effects could have arisen via three pathways. Firstly, because of the weapon condition‐dependence, selection for or against the weapon might select for better or worse quality individuals, respectively. Secondly, it is possible that the high prevalence of fighters in the population may have enhanced selection against low quality individuals unable to fight effectively. Thirdly, the high prevalence of male aggression and related mortality might have altered selection pressures acting on life‐history traits. Thus, the second and third pathways represent indirect effects of selection on the weapon, resulting from differences between treatments in the selective environment associated with male aggression. Our study was not designed to discriminate among these pathways. Instead, we aimed to estimate their joint effect, as it is the joint effect that matters for population viability and ability to endure population challenge. Nevertheless, below we discuss possible contributions of these mechanisms to increased values of fitness components we observed.

As for the first possibility, male morph determination has been shown to be condition‐dependent among acarids, with fighters arising from heavier nymphs which are able to invest in costly weapons (Radwan et al. 2002; Tomkins et al. 2011). This suggests that selection for fighters could indeed select for better quality individuals, a possibility supported in another acarid, *R. robini*, where fighters are less burdened with deleterious mutations which affect female fecundity (Łukasiewicz et al. 2020). It remains to be investigated if a similar mechanism could operate in *S. berlesei*.

The second possibility, implying that aggressive, sometimes lethal fights might have selected for better condition more strongly compared to other forms of reproductive competition, there exists some support from the study of Michalczyk et al. (2018) showing that the relationship between tritonymphal body mass (a proxy for condition at the verge of maturity) was steeper in fighter‐dominated populations as compared to scrambler‐dominated populations. However, this effect could have been confounded by population size, as fighters were more common in smaller populations, and therefore the causality of this relationship needs to be determined in future research.

Finally, changes in selective environment associated with high vs. low prevalence of fighters could have also selected for life history traits directly. Because freshly emerged males are more vulnerable to fight‐related injuries (Radwan 1993), maturing earlier than other males could be selectively advantageous. This might explain why, despite fighters taking longer to develop in both treatments, possibly as a consequence of the investment necessary for development of costly weapons, average development time across morphs and sexes was lower in fighter lines compared to scrambler lines.

Another selection pressure which likely differed between our treatments may have stemmed from an altered sex ratio, which due to male lethal combats, would be more female‐biased in fighter‐dominated populations. Female‐bias might relax inter‐sexual conflict, which in *S. berlesei* has been detected as deleterious effect of frequent mating on female fecundity (Radwan and Rysinska 1999). Furthermore, in *S. berlesei* harmful effects of frequent mating are more evident when mating takes place with scrambler males than the fighters (Łukasiewicz 2020), which could further amplify the difference in sexual conflict between our treatments. In response to relaxed conflict, females often evolve decreased susceptibility to male harm, likely due to a decreased investment in counter adaptation to male harm which results in their lower fitness when exposed to control, harmful males (Holland and Rice 1998; Tilszer et al. 2006). However, our findings are inconsistent with this prediction: when paired with control males, females that evolved under putatively relaxed sexual conflict (i.e., from fighter lines) showed higher fecundity.

We also investigated whether the prevalence of fighter males in populations affects the ability of populations to endure environmental stress. If the improvement of fitness traits we observed was due to purging of deleterious variants, we expected females and males from fighter populations to better tolerate stress. However, adult survivorship of females and males subjected to environmental stress did not differ between the treatments. This result contrasts with several recent studies demonstrating the history of more intense sexual selection is associated with better chances to survive through environmental challenge (Godwin et al. 2020; Iglesias‐Carrasco et al. 2024; but see Łukasiewicz et al. 2023). It is possible that strong selection imposed by fights in the present study selected for live fast die young phenotypes (Bonduriansky et al. 2008; Preston et al. 2011; Hämäläinen et al. 2018), and may have made them more sensitive to costs imposed by stress (Schultner et al. 2013), thus nullifying any benefits of purged genetic load. This possibility requires to be tested in future research, but we note that the evolution of shortened development time in our fighter lines compared to scrambler lines is consistent with the former representing faster life history strategies. We acknowledge that our exploration was limited in the range of environmental challenges applied, and future studies should explore a wider range of temperatures, duration of stress and other kinds of environmental challenges.

While our selection protocol was effective in changing morph proportions and in causing consequent evolution in life‐history traits, after 10 generations morph proportions stabilised. Such limits to selection are often observed in long‐term selection experiments and ascribed to the depletion of additive genetic variance or artificial selection could be countered by natural selection (Roff 1997). The latter possibility could arise in our study if the rare morph had a selective advantage. However, there is no support for such negatively frequency‐dependent selection in either *Sancassania* (Radwan 1993) or other male‐dimorphic acarids (Parrett et al. 2025).

Concluding, our study demonstrated that intra‐sexual selection favouring evolution of inter‐male aggression using costly weapons has consequence for life‐history traits potentially affecting population dynamics: time to maturity and female fecundity. Values of both traits evolved towards higher fitness values in populations selected for fighter morphs, suggesting that positive effects of intense intra‐sexual selection may overwhelm any negative effects associated with life‐history trade‐offs and/or sexual antagonism. While the mechanisms that drove evolution of life histories in response to selection in our study remain to be determined in future research, our results demonstrate that increased prevalence of aggressive males expressing costly, sexually selected weapons in a population has overall positive effects on fitness‐related traits. Further work is necessary to dissect whether these positive effects were due to direct selection on life histories under selection pressures modulated by intra‐sexual selection dominated by male aggression, or due to purging of genetic load. If the latter, future work should resolve whether it is mostly due to selection for a costly weapon or selection associated with costly fights utilising this weapon.

## Author Contributions

**Neha Pandey:** conceptualization (equal), data curation (lead), formal analysis (supporting), investigation (lead), methodology (lead), visualization (lead), writing – original draft (equal), writing – review and editing (equal). **Przemysław Piekarczyk:** investigation (equal), methodology (supporting). **Radosław Gmyrek:** investigation (equal), methodology (supporting). **Norbert Czyżewski:** investigation (equal), methodology (supporting). **Paulina Łukaszyk:** investigation (equal), methodology (supporting). **Agnieszka Szubert‐Kruszyńska:** investigation (equal), methodology (supporting). **Sebastian Chmielewski:** investigation (supporting), methodology (supporting). **Tom C. Cameron:** funding acquisition (equal), writing – review and editing (supporting). **Jacek Radwan:** conceptualization (equal), formal analysis (lead), funding acquisition (equal), investigation (equal), methodology (lead), project administration (lead), supervision (lead), validation (lead), writing – original draft (lead), writing – review and editing (equal).

## Funding

This work was supported by Narodowe Centrum Nauki (UMO‐2020/39/B/NZ8/00152/4).

## Conflicts of Interest

The authors declare no conflicts of interest.

## Supporting information


**Figure S1:** Proportion of fighter males at different larval group sizes in *Sancassania berlesei*. The error bars around the mean are the standard error.
**Figure S2:** Selection protocol for fighter and scrambler selection line. The selected populations were maintained on 14 day discrete generation cycle, and selection was carried out for 25 generations.
**Table S1:** Results from the model for selection response (change in male morph proportion) to selection for (fighter selection line) or against a male weapon (scrambler selection line) over 25 generations.
**Table S2:** Results from general linear model for fecundity of females from fighter and scrambler selection lines when paired with males from the stock.
**Table S3:** Results from general linear model for larvae to adult development time difference between fighter and scrambler selection line.
**Table S4:** Results from generalised linear model for larvae to adult survivorship difference between fighter and scrambler selection line.
**Table S5:** Results from generalised linear model for adult survivorship difference between fighter and scrambler selection line after heat exposure.

## Data Availability

The supporting data are archived in a public repository; https://zenodo.org/records/19006437.
