# Supplementary material for: Life History Consequences of the Prevalence of Aggressive Males Carrying Costly Weapons
Source: Ecol Evol. 2026 Jun 8;16(6):e73790. doi: 10.1002/ece3.73790 (PMC13243883; doi:10.1002/ece3.73790)
Supplement: Supplementary file 1 — Figure S1: Proportion of fighter males at different larval group sizes in Sancassania berlesei. The error bars around the mean are the standard error. Figure S2: Selection protocol for fighter and scrambler selection line. The selected populations were maintained on 14 day discrete generation cycle, and selection was carried out for 25 generations. Table S1: Results from the model for selection response (change in male morph proportion) to selection for (fighter selection line) or against a male weapon (scrambler selection line) over 25 generations. Table S2: Results from general linear model for fecundity of females from fighter and scrambler selection lines when paired with males from the stock. Table S3: Results from general linear model for larvae to adult development time difference between fighter and scrambler selection line. Table S4: Results from generalised linear model for larvae to adult survivorship difference between fighter and scrambler selection line. Table S5: Results from generalised linear model for adult survivorship difference between fighter and scrambler selection line after heat exposure. [file ECE3-16-e73790-s001.docx]

**SUPPLEMENTARY MATERIAL**

**Figures:**

Figure S1: Proportion of fighter males at different larval group sizes in *Sancassania berlesei*. The error bars around the mean are the standard error.


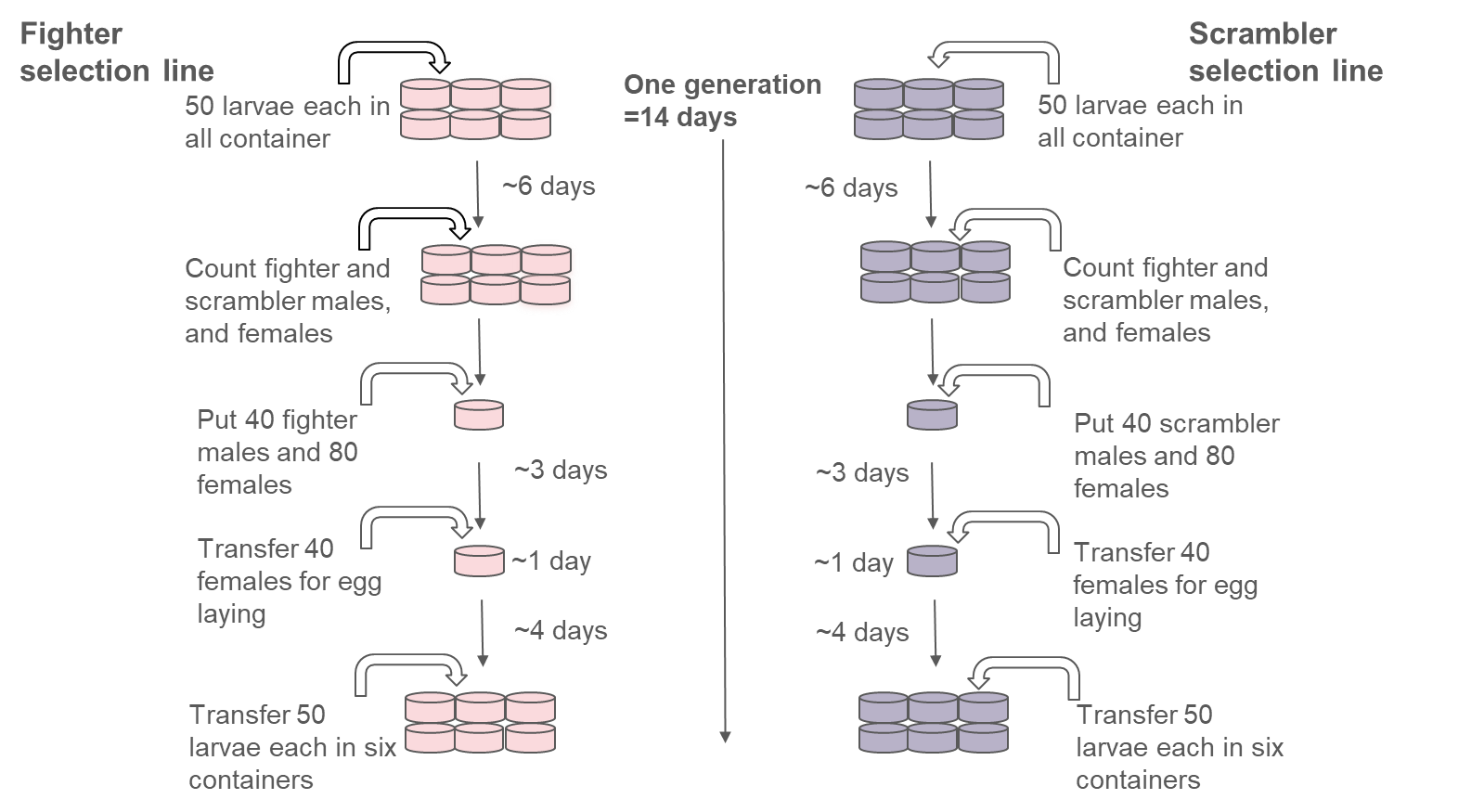


Figure S2: Selection protocol for fighter and scrambler selection line. The selected populations were maintained on 14 day discrete generation cycle, and selection was carried out for 25 generations.

**Tables:**

Table S1: Results from the model for selection response (change in male morph proportion) to selection for (fighter selection line) or against a male weapon (scrambler selection line) over 25 generations.

| Fixed effects | Estimate | | Standard Error | | Z value | | *P* |
| --- | --- | --- | --- | --- | --- | --- | --- |
| Intercept | | 1.236 | | 0.196 | | 6.280 | **>0.001** |
| Population-Scrambler | | -1.124 | | 0.276 | | -4.065 | **>0.001** |
| Generation | | 0.038 | | 0.013 | | 2.868 | **0.004** |
| Population- Scrambler × Generation | | -0.105 | | 0.018 | | -5.585 | **<0.001** |

Table S2: Results from general linear model for fecundity of females from fighter and scrambler selection lines when paired with males from the stock.

| Fixed effects | Estimate | | Standard Error | | Z value | | *P* |
| --- | --- | --- | --- | --- | --- | --- | --- |
| Intercept | | 79.464 | | 8.885 | | 8.943 | **<0.001** |
| Population-Scrambler | | -25.630 | | 10.229 | | -2.506 | **0.012** |
| Stock Male Morph- Scrambler | | -18.898 | | 10.224 | | -1.848 | 0.065 |

Table S3: Results from general linear model for larvae to adult development time difference between fighter and scrambler selection line.

| Fixed effects | Estimate | | Standard Error | | Z value | | *P* |
| --- | --- | --- | --- | --- | --- | --- | --- |
| Intercept | | 114.004 | | 1.876 | | 60.78 | **<0.001** |
| Population- Scrambler | | 5.409 | | 2.648 | | 2.04 | **0.041** |
| Sex_morph-Fighter | | 4.853 | | 2.333 | | 2.08 | **0.037** |
| Sex_morph-Scrambler | | -1.537 | | 4.154 | | -0.37 | 0.711 |
| Population- Scrambler × Sex_morph-Fighter | | 1.244 | | 4.293 | | 0.29 | 0.772 |
| Population- Scrambler × Sex_morph-Scrambler | | 11.200 | | 4.782 | | 2.34 | **0.019** |

Table S4: Results from generalised linear model for larvae to adult survivorship difference between fighter and scrambler selection line.

| Fixed effects | Estimate | | Standard Error | | Z value | | *P* |
| --- | --- | --- | --- | --- | --- | --- | --- |
| Intercept | | 1.734 | | 0.160 | | 10.783 | **<0.001** |
| Population-Scrambler | | 0.127 | | 0.190 | | 0.667 | 0.505 |

Table S5: Results from generalised linear model for adult survivorship difference between fighter and scrambler selection line after heat exposure.

| Fixed effects | Estimate | | Standard Error | | Z value | | *P* | |
| --- | --- | --- | --- | --- | --- | --- | --- | --- |
| Intercept | | -1.069 | | 0.243 | | -4.395 | <0.001 | |
| Population-Scrambler | | 0.165 | | 0.351 | | 0.471 | 0.637 |  |
| Sex-Male | | 0.801 | | 0.322 | | 2.487 | **0.012** | |
| Population-Scrambler × Sex-Male | | -0.592 | | 0.458 | | -1.293 | 0.196 | |
